# Cluster Analysis and Model Comparison Using Smart Meter Data

**DOI:** 10.3390/s21093157

**Published:** 2021-05-02

**Authors:** Muhammad Arslan Shaukat, Haafizah Rameeza Shaukat, Zakria Qadir, Hafiz Suliman Munawar, Abbas Z. Kouzani, M. A. Parvez Mahmud

**Affiliations:** 1 School of Engineering and Information Technology, University of Technology Sydney, Broadway, NSW 2007, Australia; 2 Institute for Intelligent Systems Research and Innovation (IISRI), Deakin University, Geelong, VIC 3216, Australia; 3 School of Computing Engineering and Mathematics, Western Sydney University, Locked Bag 1797, Penrith, NSW 2751, Australia; z.qadir@westernsydney.edu.au; 4 School of Built Environment, University of New South Wales, Kensington, Sydney, NSW 2052, Australia; h.munawar@unsw.edu.au; 5 School of Engineering, Deakin University, Geelong, VIC 3216, Australia; abbas.kouzani@deakin.edu.au (A.Z.K.); m.a.mahmud@deakin.edu.au (M.A.P.M.)

**Keywords:** smart meter, artificial neural network, ARIMA, smart grid, regression, SGSC

## Abstract

Load forecasting plays a crucial role in the world of smart grids. It governs many aspects of the smart grid and smart meter, such as demand response, asset management, investment, and future direction. This paper proposes time-series forecasting for short-term load prediction to unveil the load forecast benefits through different statistical and mathematical models, such as artificial neural networks, auto-regression, and ARIMA. It targets the problem of excessive computational load when dealing with time-series data. It also presents a business case that is used to analyze different clusters to find underlying factors of load consumption and predict the behavior of customers based on different parameters. On evaluating the accuracy of the prediction models, it is observed that ARIMA models with the (P, D, Q) values as (1, 1, 1) were most accurate compared to other values.

## 1. Introduction

Currently, each industrial sector is struggling because of recession due to high competition in the market. The numerous challenges that electricity companies face are price inflation, green gas emission, meet demand and supply, better customer service, and investment in smart meter infrastructure. Electricity companies are also reeling with environmental and regulatory pressures. The companies are in a constant search to look for a solution that could help them gain a competitive advantage over others. The roll-out of smart meters and smart grids have enabled the envisioning of the future of different power companies. However, there are still miles to go before actually attaining the full potential that these latest technologies can bring. The current market has seen a big boom for data growth in many different sectors, such as IT, biomedical, transportation, and the share market. Data analytics carry great potential in unveiling future benefits that we cannot simply think of or find.

Companies have been trying hard to find ways to help them overcome relevant issues, but more challenges come up related to the changing behavior of customers, the demand–supply cycle, investment in AMI, and asset management for the industry. Companies are diving into the well to overcome these problems. Predictions to gain insights into the future of smart grids in order to invest, plan and make important decisions can be made through data mining. The basis of forecasting is to unfold the consumption pattern of customers and try to build a system around that. Utility companies try to observe and understand the usage and consumption of customers based on different factors and try to uncover potentially hidden aspects of their future growth. For example, the consumption of energy, on an hourly, daily, weekly basis, is recorded, and then corresponding changes in consumption during peak hours are noted. In addition, many other factors, such as weather, demographics, and economic conditions, are inculcated for better and accurate decision-making [1]. To gain insight into the future of smart meters, the Australian government also ran a trial project named Smart Grids Smart City (SGSC). They set up smart meters for the residential areas of Australia. They collected enormous data about usage over three years. The way to gain insight into the various challenges lies in the data that have been collected from the customers. The data collected have a large number of attributes, and drilling down the data through data mining can certainly give many answers to help get predictions for the future. The purpose of this paper is to address the benefits that can be attained with the analysis of the collected data. Both statistical and mathematical models can be found in many literature reviews for load forecasting. For instance, an artificial neural network is very good in non-linear computing patterns, ARIMA and regression models being popular for linear calculations. Time-series forecasting has proved to be a computationally expensive task in the case of load forecasting as it becomes extensive to apply it to each customer. Models based on computational intelligence are, thus, needed for efficient analysis of temporal data. To propose a solution to this issue in the case of load forecasting in smart grids, this study has selected three less expensive models from the literature in terms of computation. These are ANN, Auto Regression, and ARIMA. These models are applied for load prediction in smart grids, and the best one among them has been determined by analyzing the accuracy of the results. The method ARIMA has shown the best performance outcomes with the highest accuracy. In this paper, we try to load forecast for a short period, i.e., summers in New South Wales, Australia, for the customer usage of electricity using different data mining algorithms, followed by estimating their accuracy and finding the best model among them. The paper will also elaborate on clustering the customers using K-means depending on their consumption pattern, analyzing the pattern, and making predictions based on profiles, work habits, and economic status. This would assist in potentially answering some questions that were hidden in the data and in addressing factors that affect the load forecasting for smart meters [2].

## 2. Literature Review and Problem Relevance

**Figure 1:**
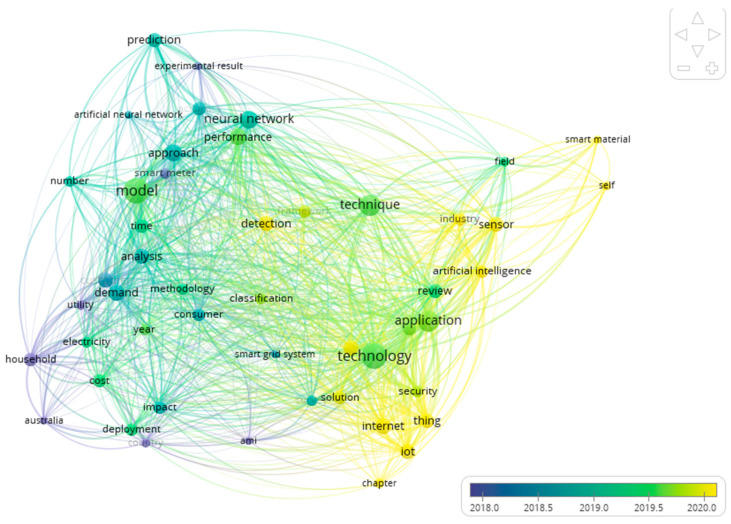
Keywords clusters using VOS Viewer.

Time-series forecasting is a big issue for energy utility. It is not a good approach to apply prediction for each customer, as it is more computationally expensive. There are different ways to solve and address this problem. Through the literature review, we will find the best possible solution to solve this problem. We chose the websites of Web of Science, IEEE Xplore, and Elsevier to retrieve the articles from the best journals and conferences. To find the most relevant research articles from the recent literature, we first formulated a set of basic keywords that are to be entered into the search engines of the websites. These keywords include “Smart meter”, “Artificial Neural Network”, “ARIMA”, “Smart grid”, “regression”, and “clustering”. Using VOS viewer software, the most used keywords found in the recent literature, which are relevant to the basic set of keywords, were retrieved. The clusters of keywords retrieved using this process are shown in Figure 1. These keywords were used to form search phrases, which were then used on the search engines of the selected platforms. The yielded search results consisted of ranked research articles based on their relevance to the entered search phrases. All the research papers have been published within the past decade and are written in the English language. After downloading the articles, the content of each paper was carefully examined and analyzed to confirm its relevance to the problem domain.

In the paper [3], the author showed the importance of clustering for distribution network operators. It can help in finding the candidates for demand response and segment customers as per their usage. He recorded the intra-day consumption of different groups of customers into four intervals. Using the Finite Mixture Model, he clustered the customers in ten different groups based on a similar consumption pattern [4]. Those clusters represent different behaviors of customers. In the end, the clusters were double-checked for their reliability and robustness through the bootstrap technique [5].

In this published literature, the aim of the author is to improve the accuracy of forecasting electricity consumption at a system level, depending on the behavior of the customers by grouping them and extending the predictions to the system level. He explained that many researchers have given estimations and predicted load forecasting at the system level without the use of information from lower levels, such as the household, feeder, and transformer level. This reduces the data size and reduces the additional computation load of the system. The reason for such predictions is the lack of efficient data collected from lower levels. He gives a three-step approach, starting with the clustering or grouping of customers with similar consumption patterns and then predicting the consumption for the group of customers; then, from the group consumption level, he predicts and moves to the system level, using actual data from two electricity companies in the U.S.A. K-means clustering was used to cluster customers and their smart meters depending on their consumption behavior on different dates and formed groups of customers. Then, those summed smart meters collectively obtained the system load forecast [6].

Reference [7] performed research based on giving short-time load prediction on the real data of residential customers that were provided by a utility company, using spectral analysis and Kalman filtering. They used sample data as the subset of the original data in order to prevent high computational power. They had two attributes, i.e., weather and lifestyle, to make the prediction. Using the sampling data, they made certain predictions. They also evaluated the accuracy of the prediction using different sampling and test data. It is known that with more real-time data, the accuracy of the prediction increases, but also, the computational cost increases [8]. So, we have to carefully select the sampling data in order to get the desired level of prediction but through a lesser volume of data and less computation cost. Based on the results, the authors concluded that by reducing the data volume, the additional computational load could be avoided without having any effect on the anticipated performance of the system. However, this requires careful selection of the sampling rate of data. The author tried to differentiate between the statistical and artificial approaches for load forecasting based on better interpretability and accuracy. Through multiple linear regression, he forecasted the load for a U.S. utility company, using factors such as temperature, day, hour, and month for various purposes, such as Demand Supply Management (DSM), planning, and the purchasing of electricity. They proposed a regression model, which predicts hourly forecast for three years.

The author in [9] describes the parameters working behind demand forecasting. He reports that forecasting depends on many attributes, so there is no one size that fits all kinds. He uses different attributes, including climatic conditions and historical data, in the prediction models, such as artificial neural networks and auto regression. He finds ANN and AR to be very effective, as they can improve the accuracy of the prediction and do not require many parameters for prediction. Using three different terminologies (i.e., short term, midterm and long term), predictions can be forecasted, and the various benefits of these predictions are discussed. In this paper [10], the short-term load forecasting for utility companies is described, along with the concept of data processing and the designing of the neural model with different attributes, such as wind, temperature, and humidity. The authors conclude ANN to be very effective, even with very few parameters. They suggest that, with more parameters and attributes, its accuracy could be enhanced further [11]. This would significantly help in reducing the computational load of the system. Furthermore, ANN is a model based on computational intelligence; hence, it is one of the best methods to target the problem of excessive computational burden.

In paper [12], the author used an artificial neural network to predict short-term load because of the non-linear characteristics of ANN compared to other statistical models such as ARMAX, ARMA, regression, and Kalman filtering. Using MATLAB, he did a comparative analysis of six different models of ANN based on their characteristics, performance, and simulation time. Further, he proved that a radial basis neural network is the most efficient and fastest approach with minimal simulation time and fewer errors. The author proposed an approach to minimize the number of hidden neurons in the ANN model. He also suggested using a radial basis neural network, along with his proposed approach, to get the best outputs and prediction for short-term load forecasting.

In literature [13], the machine learning model, i.e., Artificial Neural Network (ANN) is compared to the statistical model, Autoregressive integrated moving average (ARIMA), for short-term load forecasting for a Dutch residence. This research study is carried out for a one-day-ahead load forecast for the residential scheduling storage of energy. Their predictions were only dependent on the electricity consumption of a household, which was based on time; so, it was a time-series forecast which had linear as well as non-linear consumption parts. ANN supported the non-linear, and ARIMA contributed to the linear part. Overall, they found ANN to be better in predicting a day-ahead load for the house.

In paper [14], the author identified the best ARIMA model for load forecasting. Two different data sets were used: one for consumption and the other for transmission. By using these models, they compared the values for the year 2010 with the actual value. Later, they choose the best model for the prediction of time series based on the result that was close to the actual values [1]. ARIMA targets the problem of excessive computational loads by producing accurate results, even when a small size of prediction data are available.

Table 1 presents a comparison of the results of the studies reviewed in this section. Authors in [3] worked on smart grid data, using a clustering approach. Their results demonstrated that the demand behavior of customers depends on seasonality and weekdays. Therefore, these two factors behave as sources of variation. Authors in [7] used Kalman filtering to predict the load of customers residing in a residential area. Their results showed that MAPE was significantly reduced when the sampling period was reduced from 30 min to 15 min. Authors in [9] used regression methods and ANN to forecast the demands of the customers in smart energy grids. According to the results, increasing the number of layers in neural networks does not cause any improvement in performance. Therefore, only one layer was used in the hidden layer. An improvement in the prediction results has been recorded with ANN and regression methods. Furthermore, by introducing a compensation factor in the load profile data, the performance outcomes have been further improved. Authors in [12] used ANN to perform load forecasts. They demonstrated that the radial basis neural network is the fastest and most accurate model of neural networks for load prediction. In addition, the number of neurons in the hidden layer must be as little as possible to overcome the overfitting problem. Authors in [13] proved that ANN provides much better performance results than ARIMA for load forecasting. This is due to the additional failure of the ARIMA method to calculate the peak size. The author in [14] used ARIMA for load prediction and found it to be accurate in forecasting the consumption of load and its transmission in the province of Punjab, Pakistan.

## 3. Smart Grid Smart City (SGSC) Project

This study used customer trail data from the Smart Grid, Smart City (SGSC) project. It is an initiative by the Australian government that started in late 2010. Ausgrid led, along with several other organizations, in order to analyze the reliability, cost, benefits, and other effects of smart grids to manage their usage, to have insight into its growth, to frame policies for smart grid usage, and to address various business cases, with a budget of AUD 100 million [15].

### 3.1. Smart Grids

A smart grid is a power grid that utilizes operational and energy measures, such as smart meters, smart appliances, and other efficient methods of communication in order to respond quickly to the changing demands and control the production and distribution of electricity [16]. Why are smart grids smart? The formation of smart grids has changed the way traditional grids used to work [17]. Smart grids have a focus on providing energy reliably, efficiently, and sustainably. They have empowered customers by helping them to monitor their usage, manage their bills, and reduce energy costs, and minimized the load on utility companies, decreasing burnouts and blackouts and supporting renewable resources [18]. Some of the factors that make them smart are as follows:

- Supports renewable power technologies—Unlike traditional grids, the integration of distributed renewable energy was very challenging because of its unpredictable nature. For example, energy from the sun and wind energy from windmills are irregular and unsteady. In addition, they are geographically separated. Therefore, the energy from these sources needs to be stored by smart grids for future supply.
- Ease of managing and troubleshooting brownouts and blackouts—With smart grids, if there is a power surge, blackout, or any other problem, it can be easily identified remotely and can be solved locally. There is no need to go and collect information from the location site.
- Empower customers to control—Smart grids have empowered customers to manage their bills efficiently. It has given more control and real-time information to the customer about their usage so that they can have a better understanding of their usage and can control their bills.
- Reduces utility expenses—The customers have better knowledge of their consumption patterns and are aware of peak hours. They can reduce their consumption during peak hours. As a result, it will help utility companies in saving the money that they spent on standby plants, which are only used during peak hours and stay idle the rest of the time.
- Better prediction for tomorrow—Smart grids can efficiently predict the increasing demand and help them to meet the supply for the future. In addition, it gives an insight to many new future aspects.

### 3.2. Smart Meters

A smart meter is a device that records electricity consumption for a short interval, generally every half an hour, and enables a two-way communication channel with the electricity distributor to share the reading in order to monitor and make informed decisions on the usage, end estimated electricity bills, and overcome the traditional way of manual meter reading. Capabilities of smart meters [19] include the following:

1. The rollout of smart meters has ruled out the need for manual meter readings and expected estimated bills. Bills are now more accurate. It also saves on the time and money spent on sending a person to take the reading.
2. Flexible tariff plans are available by the electricity distributors in order to reduce their peak load. The tariff is designed with different pricing at different hours of the day. Customers can save their bills by changing their usage patterns.
3. People are trying to invest in solar energy because of smart meters. Further, they have an option of producing and selling their energy to the distributors.
4. Using in-house panels, customers can observe and manage their usage patterns.
5. It facilitates the customer in case they have to move out of their house. The supply can be remotely triggered, and the services are very cheap.
6. Use of electric vehicles will be available.

### 3.3. Forecasting in Smart Grid

Forecasting constitutes getting an insight into the future, which can be helpful in making wise decisions today for a better tomorrow. Load forecasting for an electric utility is useful in managing the demand and supply of power. It affects daily operations, such as planning of resources, fuel asset arranging, and making vital decisions. Load forecasting has been acknowledged for its predictions that could help in the economic and strategic growth of the company. The accuracy of forecasting is of vital importance [20]. With an increasing demand for AMI and implementation of smart grids, load forecasting, and the addition of distributed sources of energy and renewable power generation, it can be very useful to the companies in predicting the future growth areas. It can help predict major demand sites, identify and profile customers, help them to better formulate their tariff plans as per the needs of the customers, enable them to meet the power supply, and guide them about the future growth rate. Forecasting could be long term, for years, midterm, ranging from weeks to months, and short term, ranging from several hours to days.

### 3.4. Factors Affecting Forecasting in Smart Grid

With the changing demand, customer behavior, sources of energy, increasing data, and other endless parameters affecting load forecasting, it has become a challenge for companies to get accurate predictions. As discussed above, load forecasting has enormous benefits. To realize those benefits, we need to accurately design the models, as the customer is now becoming aware of their power usage, along with all the factors and parameters in consideration that can affect the prediction. There is no one size that fits all models. It is a constant strive to meet the best model. The following are some of the factors that can affect the prediction.

Firstly, the usage of smart grids records data at a very granular level with time intervals ranging from 15 min, so this high volume of data is not easy to store, analyze and make predictions with. Secondly, there are several customers that have both traditional and smart meters installed on their premises, so accurate forecasting is a challenge. Thirdly, the difficulty in identifying and quantifying all the parameters affecting the forecasting and further unavailability of data from different perspectives, which affects the demand and prices of electricity, poses challenges to the forecasting system. Lastly, the accuracy of the prediction models that have been used to conceive the results affects the prediction.

## 4. Proposed Methods and Materials

From the knowledge acquired from the literature review, it can be seen that it is challenging to find the best model for load prediction. Therefore, we will be using the models mentioned in 9 and 13 on our data set. Many factors and parameters govern forecasting. Before proceeding further, it needs to be assured that the data to be used is clean, good, and relevant without any noise, ambiguity, and incompleteness. To get confidence in the data to be used, the data have to be prepared and cleaned. ETL (Extraction Transformation Load) is one of the important steps that govern the outcome of any model. From the literature, the importance of data preparation and clustering and how it can affect the overall performance of the system is evident. Based on the models discussed in the papers, we have found three models that we will try to implement in our case to find the best model among them.

### 4.1. Implementation

The steps involved in making a model for prediction purposes are as follows:

1. Preparation of data—After getting access to the data, it is important to extract, transform, clean and load the data in the database.
2. The second step is to find the most suitable model, calibrate according to the attributes, and execute.
3. The third step is to visualize the data, gain insights and make informed decisions.

### 4.2. Data Preparation

The original data contain 10 attributes and were recorded for a duration of 3 years. The total size of the dataset on the Australia website was 16.5 GB in an uncompressed form, and 11.59 GB in a compressed form; the dataset contains 311,468,580 records. The attributes under use are from the downloaded data for the purpose of analysis:

- Customer_id: Represents the customer in the data set.
- READING_DATETIME: Shows the date and time when the reading was captured.
- GENERAL_SUPPLY_KWH: Electricity consumption in kilowatt per hours.

**Figure 2:**
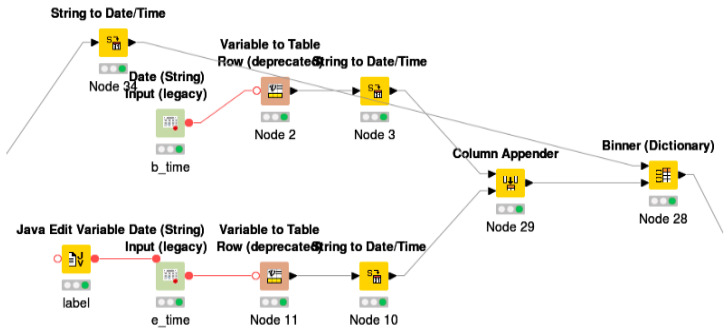
Sub-workflow for time window filter Meta node.

File reader node was used to read the data, and column renaming conventions were used to rename the data more appropriately. For instance, the column General_supply_Kwh was renamed to KW/30. As our focus was to get the seasonal summer data (from December to February), the date range was defined in “time window filter” Meta node as shown in Figure 2 and Table 2.

To get the electric consumption at the customer level, for example, to get the usage at certain date and time, the date field extractor node was applied on the date and time fields, respectively [21]. The purpose was to get separate fields into year, month, and days for the date and hourly and minute values for the time. The separation of fields in date and time order was done with the aggregation of the kw/30. With this, we are able to divide the customers with different time-series values. For example, we can see the hourly, daily, weekly, monthly, or yearly electricity consumption for a specific customer.

To create the hourly segments simulation, we used the simple rule in the engine, i.e., if the hour value is greater than or equal to the specified threshold, then we assigned it to the specified range, and vice versa. At the end of the data preparation stage, we were able to decompose the data further and extract information, such as the total consumption of a single customer, average daily values, and calculation of percentage according to hours and days in a week. Different analyses can be made on each customer level, and predictions for future usage can be obtained, but because of the large data generation, this approach would not be productive and effective, especially at the grid level.

### 4.3. Clustering

To bridge the gap between the customer and the grid, the best possible solution, as suggested by [3], is to group the customers with similar electric usage behavior. The clustering approach and hybrid concept are interesting, as mentioned in paper [22] with the concept of the danger theory. To identify the users who are consuming the same amount of energy or, in other words, check their likelihood, those who have the same pattern will be combined. This information is useful especially for retailers to identify the customer who shares the same behavior. The profiling of customers can be used to provide them with better tariff rates [23].

The objective of applying this algorithm was to group together and profile, based on the premise of normal qualities and day-by-day and week after week, appropriation estimations of the power use. The elements accessible were as follows:

- Average and percentage of the utilized vitality on every weekday from Monday through Sunday.
- Percentage and average rate estimations of power utilized amid five distinctive day segments.
- The aggregate power utilized over the test time as a part of kW.
- The average yearly, month to month, week after week, day by day, and hourly utilized energy as a part of kW.
- The energy used throughout the weekend (WE) and over business days (BD).

The aggregated percentage values from the previous section were used for clustering. To accommodate the maximum number of customers to their behavioral measures, the Elbow and Silhouette methods were used to determine the value of K (29). Since the K-means algorithm is a distance-based method, all columns need equivalent ranges, that is, they should be standardized. A “Normalizer” node was presented, and a “min–max Normalization” into the [0, 1] interim was connected on all information segments [24]. After extracting the features of each cluster from the K-means model, the next task was to build the average time series for each cluster. This was achieved through the “time series” Meta node. The working of the Meta node is shown below.

**Figure 3:**
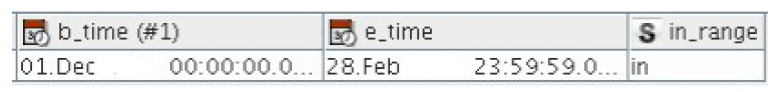
Data extraction range set on the input legacy node under time window filter meta node.

**Figure 4:**
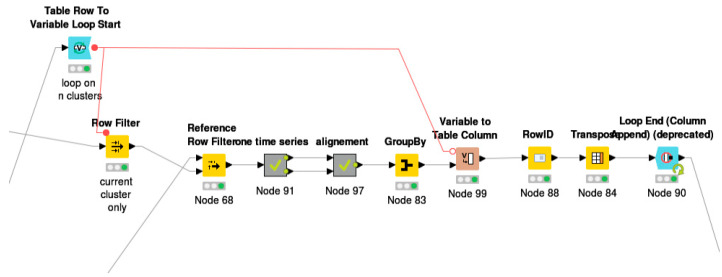
Sub workflow for time series Meta node.

Further, to obtain the time series for each cluster, the hourly, daily, weekly, or monthly time series from the previous section were filtered and fed into “time series” followed by the “alignment” Meta node, as shown in Figure 3. The loop node was implemented to extract the values for each meter with respect to its assigned cluster as shown in Figure 4.

## 5. Case Study (Business Case)

The data from the trial of SGSC could possibly shed light on a variety of applications. For example, it can be used to cluster and profile customers with a similar type of consumption, formulate new tariff plans, demand response, forecast load, invest in AMI, and address customers about their usage patterns and their contract, and seek future growth. This paper focuses on the cluster visualization, an analysis based on consumption patterns, and prediction models’ comparison.

### 5.1. Stakeholder

The major stakeholder of knowledge gained by cluster visualization and load forecasting will be the electricity manufacturers, distributors, or utility companies. They can take advantage by analyzing these clusters and using them as a knowledge base for making their future decisions and plans. The energy exchange markets and end customers will also be indirect stakeholders, as the knowledge gained by the utility companies will affect their plans, which in turn will affect customers. For example, the new tariff plans made will affect the customer, only. Further, all the sectors and people related to the electricity company, the academic people and researchers could be tertiary stakeholders.

### 5.2. Clusters Visualization and Analysis Based on Consumption Pattern

Cluster visualization can be an important aspect for utility companies. It can enlighten them about various dimensions for analyzing usage patterns of consumption to have more knowledge about their customer. They can gain knowledge about their behavior from their usage patterns. Different clusters can be used for comparative analysis, help in segmenting different profiles of customers, gaining new insights.

From our dataset and by susing K-means clustering, we have formed ten clusters for a different group of customers based on their consumption pattern. We have identified their sizes and their usage for the summer season. Comparing different consumption criteria patterns and usage, we can assume the social, economic, and work profiles of these families. We can further use this information to classify customers and form tariff plans as per the needs of the customers.

#### 5.2.1. Cluster Segmentation Based on Size

The data taken are three months’ data (December–February) from the Smart Grid Smart City trial project. Let us visualize it and try to analyze the clusters in terms of their electricity consumption from different perspectives, such as daily, intraday, weekly, and monthly consumption and that for the summer season. Here, from the gathered data, it can be seen that some clusters are formed with a small size, such as cluster 6, which is the smallest one that accommodates 145 houses, while there are big clusters, such as cluster 9 that contains more than 2000 houses that share a similar pattern.

#### 5.2.2. Summer Season Load Consumption of Different Clusters

**Figure 5:**
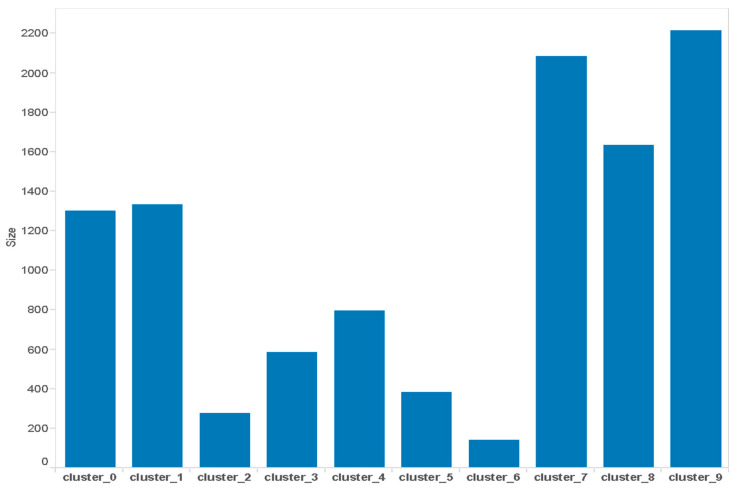
Bar chart for clusters according to size.

Total power consumption of different clusters over three months during the summer (December–February), corresponding to their size, can be seen in Figure 5. The following are some of the assumptions we can make from these clusters. Firstly, clusters 3, 4, and 6 are very small, and their consumption rates are very high, especially cluster 6. Therefore, this makes us wonder about the possible reason for such high consumption. There are a few parameters that we have found which could be the reason. These clusters could be big families with more rooms in the house that have more people in the families. A second reason could be that in these families, most of the time, people stay at home and use appliances. Therefore, the work profile of these people could be that of retired or older people, and they must be having some business. As we have seen in the data for summer, we can possibly assume that they use air conditioners in all rooms. Their economic condition is very well such that they afford such high consumption.

**Figure 6:**
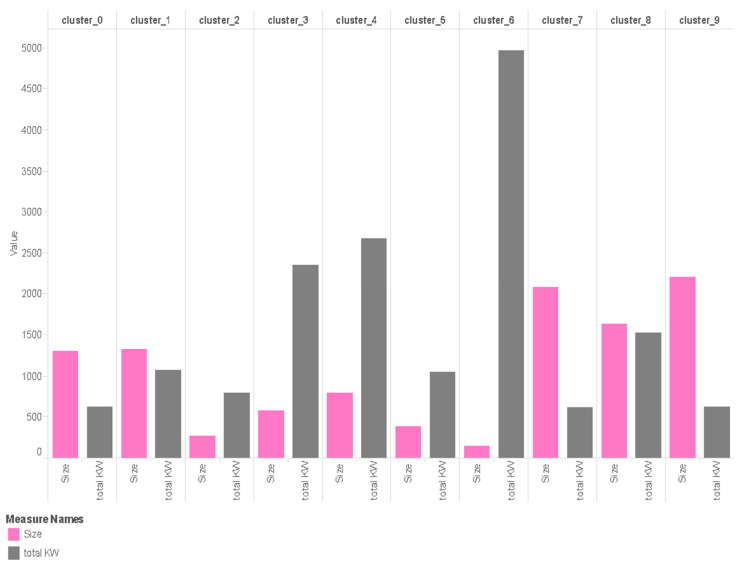
Bar chart for cluster’s total electric consumption according to size for summer.

Another set of clusters is 1 and 8. It is, again, a big set of clusters, and they have neither too high nor too low consumption, so we can assume that these are middle-class families of 4–5 people in the family as shown in Figure 6. They could be working families, and they might have some older adults who stay at home, as they have moderate consumption. Moving to clusters 2 and 5, they have very small sizes, but their consumption is between moderate and high, as per their size.

So, it is probable that they lie somewhere in between middle-class and high-class people. Their family size could be small. Looking at clusters 0, 7 and 9, we find that these are one of the largest clusters in size, but their consumption is one of the lowest. Therefore, we can assume that these are working families, probably a couple or a one-child family who are working. They do not use many appliances at home, as they do not stay at home much.

#### 5.2.3. Monthly Consumption of Clusters

**Figure 7:**
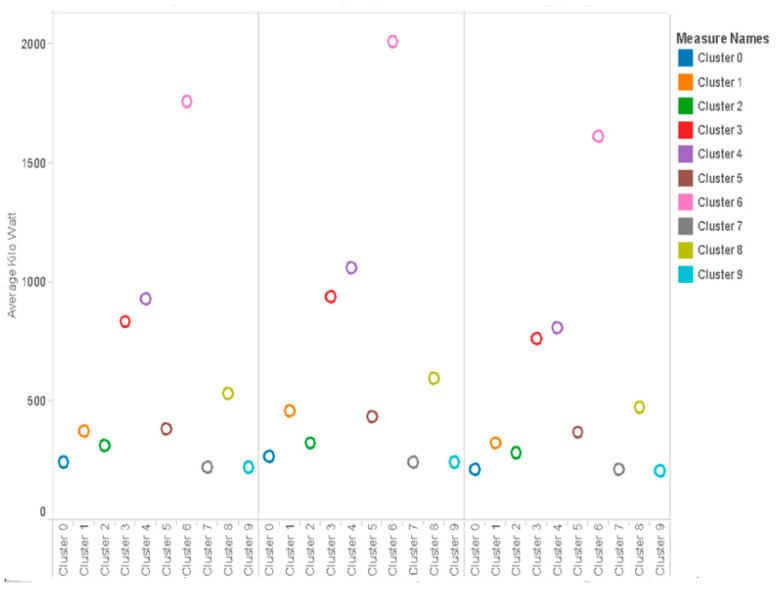
Average monthly kW for clusters.

The monthly consumption of electricity of ten different clusters over three months (December–February) of summer is as follows (Figure 7).

From the monthly consumption, we can analyze that approximately all clusters show an increase in consumption of electricity in the month of January as compared to December and then show a fall in February. This is possibly because in January, every family is back from holidays and has resumed their work. In addition, the summer is at its peak in January, so there could be an increase in usage of air conditioners. The temperature is decreased in February, so overall consumption is also plunged.

#### 5.2.4. Weekly Consumption of Clusters

Weekly electricity consumption is over the month of December by ten different clusters. It can be analyzed that the consumption pattern of clusters weekly in the month of December, where we find that by the third and fourth week, i.e., Christmas and New Year, most of the clusters show a little fall in the consumption. This is probably because they are on holiday or are busy shopping.

#### 5.2.5. Consumption on Weekdays vs. Weekends

**Figure 8:**
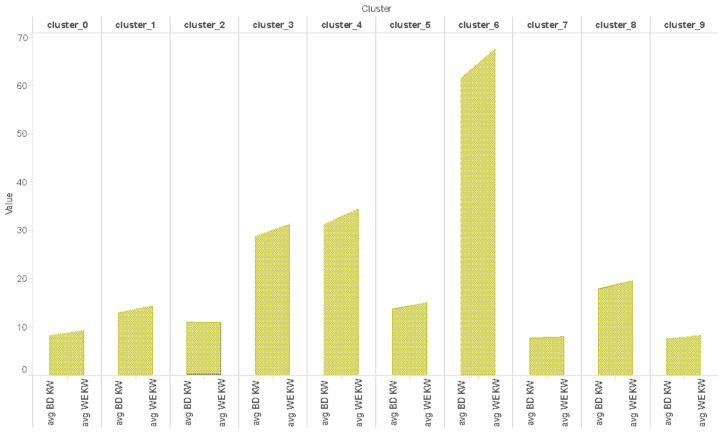
Average usage on weekdays (Monday to Friday) and weekend (Saturday and Sunday).

The electricity consumption of ten different clusters over three months (December–February) of summer, in terms of the consumption on weekdays vs. weekends, where BD stands for Business day and WE as weekend, are as shown in Figure 8. One thing clarified here is that if we look at the consumption depending on the day, all clusters except clusters 2 and 7 show a rise in consumption on weekends. This is because everyone is at home on weekends, which means that more appliances are used, hence, more consumption. Overall, there is more consumption on weekends, which means more load on the utility companies. Therefore, this can help them to calculate how much extra load is expected on weekends in order to take appropriate actions. Clusters 3, 4 and 6, that have more consumption on weekends, could be segmented separately and offered special tariff plans. From this consumption, we can also assume that clusters 2 and 7 are families working on weekends as well.

#### 5.2.6. Daily Consumption of Clusters

**Figure 9:**
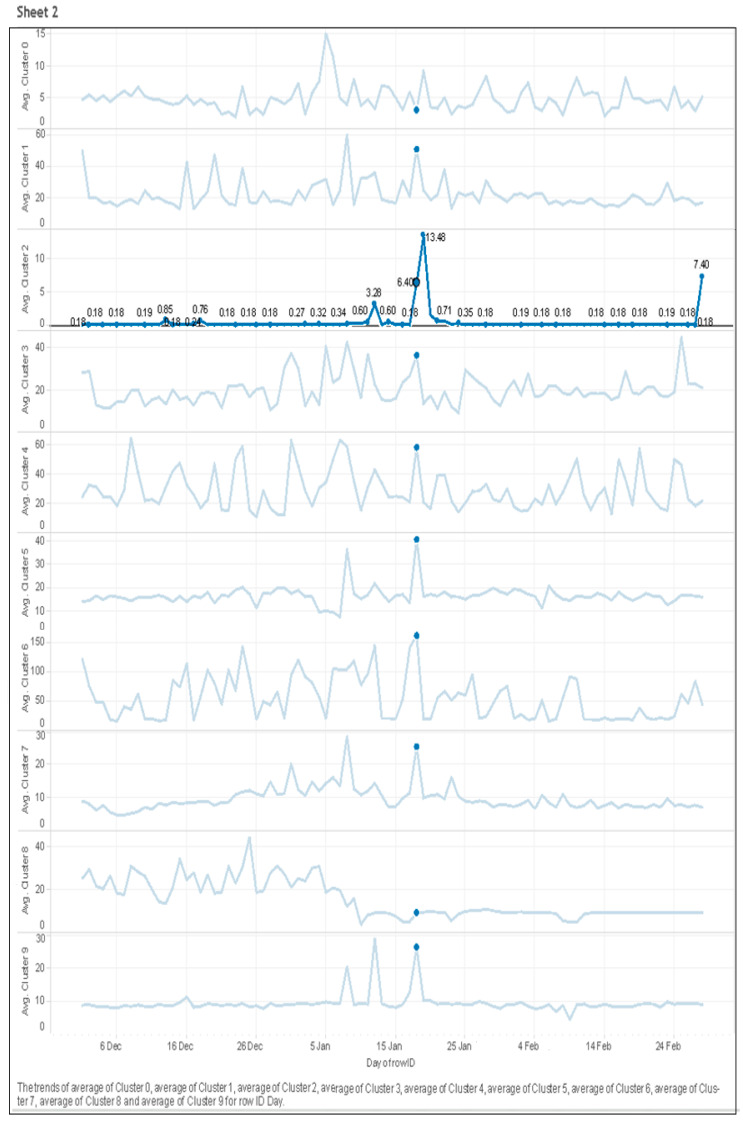
Average daily usage of clusters.

The line chart represents the electric consumption by ten clusters over three months (December–February) of summer in a daily fashion as shown in Figure 9. Cluster 2 suddenly shows the peak value. As we already had performed the data preprocessing, there was a minimal chance for data noise. It is interesting to see that this peak was not only the case with cluster 2, but the rest of the clusters were also showing the same pattern, which makes us more curious. On our investigation, we found that this trend was due to the high temperature of 46.5 degrees Celsius that was marked as the hottest day by the Bureau of Meteorology [25].

#### 5.2.7. Intraday Consumption of Clusters

Table 3 represents the electricity consumption by ten clusters in five different hourly intervals of a day over three months (December–February) of summer. Consumption is defined as a percentage of their total consumption. For intraday consumption, we divided 24 h in 5 intervals according to the consumption pattern, which are as follows:

From the consumption pattern, we can visualize that most of the clusters have maximum consumption in the 7–21 h interval. The reason probably could be the use of air conditioners at nighttime. The second most consumption time is 17–21 h. We can assume that people are back from work, and this time is spent bathing, cooking, etc. Most of the electricity could be used in the kitchen and bathroom. Further, we can see that clusters 3, 5 and 9 have the highest consumption percentage at nighttime, which possibly means they use air conditioners throughout the night. They have very little consumption from 7 in the morning until 17 in the evening, which means they could be working families and are not at home during this interval. Clusters 1 and 2 have less consumption at nighttime even though it is a big interval, which might mean they could be working late at night and their consumption is higher during the day, which also means they use most of the power during the daytime. Moreover, clusters 1, 4, 6, 7 have equal amounts of consumption in the morning and day and are a bit high in overall consumption, which predicts that they have big families or are retired people who stay at home throughout the day [26]. The energy consumption and demand reach their peak during the summer season, which accounts for the noticeable peaks in the results.

From the above visualizations and exploration, we made some assumptions from the data interpretation. The paper focused on different parameters, such as temperature, holidays, weekdays, weekends, economic, social status of residential. We could explore a lot more information from these clusters, and the utility companies can use these for various purposes. Depending on these visualizations and interpretation, we can figure out the work profiles of the people. Learning from the consumption by the aforesaid characteristics, we can manage the demand–supply of electricity, profile customers, and design or customize tariff plans as per their needs, which will not just help the customer save on their bills but also help utility companies save power during peak hours. Understanding these clusters and interpreting them can be very helpful. The forecasting of these clusters can give insight for future work; the attributes identified can be part of prediction models for the companies to make more accurate predictions so that their businesses can flourish.

## 6. Results and Simulation for Models for Time Series Forecasting

The data were trained from the previous 120 h time-series data to predict the next 120 h data. Table 4 shows the comparison of the dataset used in this study with the dataset used in previous studies that used smart-grid data in the past decade. Authors of [27] used data from a period of 1 year. These data had five attributes, and a K-means clustering technique similar to the one proposed in this study was used. In [28], the authors worked with 1.5 years of data containing 81 attributes. A data normalization step was included to pre-process the data. This involved applying the zero mean and unit variance-based method on the data [28]. In study [29], 29 features were included in the dataset. A feature extraction approach was used in the data pre-processing step. By reducing the number of features from greater than 100 to 29, it was demonstrated that the prediction accuracy of the system increased considerably. Authors in [30] used data from a period of two years and applied data filtering steps to take data only from weekdays. The comparison shows that this study uses data from a larger time duration than previous studies. Apart from that, the applications of two pre-processing steps, such as K-means clustering and normalization, further refines the data and prepares them to be efficiently used by the prediction models. Clustering performs an important function in finding appropriate candidates to determine the demand response of customers. It also improves the modeling of the energy profile [3].

### 6.1. Artificial Neural Network

This is a machine-learning algorithm inspired by a biological neuron network. Similar to the neurons in the brain, it is a model of interconnected nodes, which exchange messages with each other, and is used to form new functions based on the inputs. The nodes or neurons are capable of learning, and the connections between nodes have some numeric weight, which increases with learning and experience. It has vast applications and is very useful where rule-based programming does not work. It has been widely used for forecasting electric load. They are non-linear circuits and can perform non-linear curve fitting. Their output could be either linear or non-linear mathematical expressions.

#### 6.1.1. Results for ANN

As the neural network takes the input in the nominal form, the normalization was applied to the input and with the help of partitioning the node, the input data were split into two sets. The neural network was trained on 15 hidden neurons per layer that resulted in a 0.003 mean square error.

**Figure 10:**
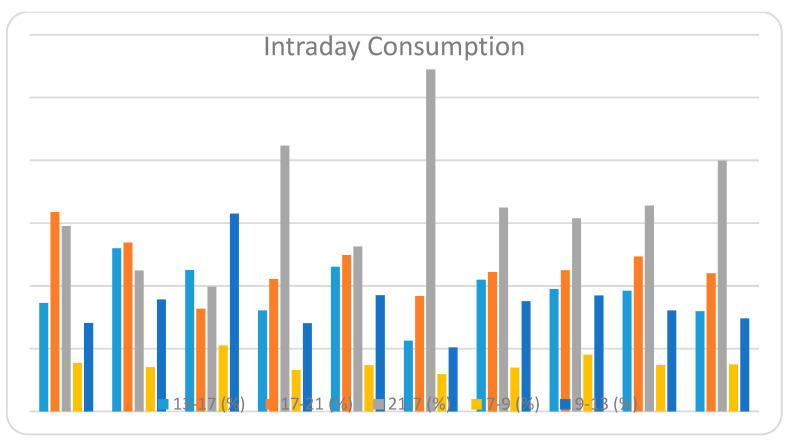
Intraday consumption.

In Figure 10, the prediction of the 120 h on actual values was calculated through a neural network. The blue color represents the actual value, while the red color represents the predicted values. The *y*-axis represents the usage while the *x*-axis shows the time in hours. The prediction is for 120 h.

#### 6.1.2. Auto Regression Model

**Figure 11:**
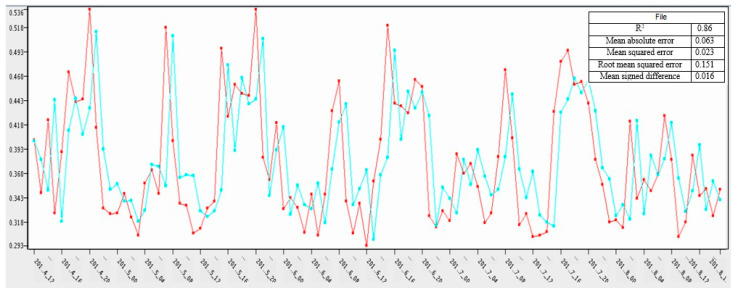
Output for statistics node and graph for AR (i.e., blue colour represents the actual value while the red colour represents the predicted values).

The auto regression (AR) model represents an irregular procedure, which shows nature and financial matters over a certain time in Figure 11. The autoregressive model determines that the yield variable depends directly on past qualities and on a stochastic term (University, 2016).

**Figure 12:**
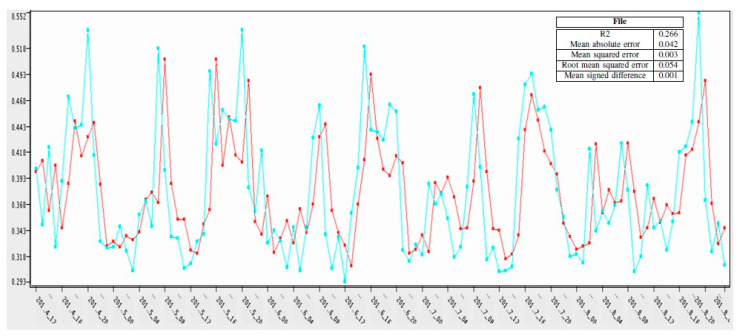
Output of statistics node for ANN and neural network (i.e., blue colour represents the actual value while the red colour represents the predicted values).

In research, the auto regression model was trained with 95% data to get the prediction for the remaining 5%. The calculated mean square error was 0.023. In Figure 12 the *x*-axis represents the time in hours (120 h), and the *y*-axis represents the predicted values on the actual value for the same time.

#### 6.1.3. ARIMA Model

**Figure 13:**
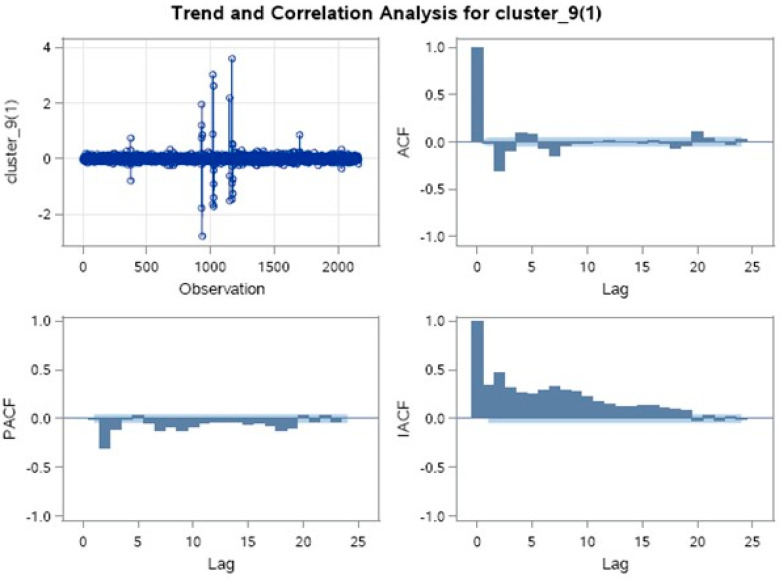
Trend and auto-correlation for ARIMA (1, 1, 1).

The ARIMA model is a unique type of regression model with dependent and independent variables. The dependent variables are fixed, and free variables are all slacks of the reliant variables or slacks of the blunders, as shown in Figure 13.

It is clear on a basic level that an ARIMA model can be extended to fuse data given by driving pointers and different exogenous variables by the addition of one or more regressions to the estimating condition. It is an extension of ARIMA to a non-stationary process and is mostly used for time-series prediction. The ARIMA model can be denoted by the following equation [31]:

(1) $${\hat{Y}}_{t}-\varphi_{1}Y_{t-1}=\mu -\theta_{1}e_{t-1}$$

where ${\hat{Y}}_{t}$ is the *t*th time series load data; *Y_t_*_−1_ stands for (*t* − 1)th time-series load data.

As discussed in the paper [14], we are going to implement the ARIMA model in SAS with different P, D, and Q values, where

- P represents lag or delay of unvarying series in the prediction equation, also called auto regressive order.
- D represents a count of differences required to bring stationary, i.e., difference order.
- Q in the prediction equation is the count of moving average terms or the number of delay prediction errors i.e., moving average order [32]. The dependent variable was set to cluster 9, while time-id was set to the series time-id.

As the ACF (autocorrelation function) over lag is not gradually decreasing, this shows that the values are stationary and do not show any kind of seasonality, such as hourly or daily, etc [33,34]. To choose the best model, we used two tests, AIC and SBC, and applied them over individual ARIMA models (Table 5). The Akaike information criterion (AIC) is a basic set of data that is used to determine the relative value of the statistical computation. Therefore, the given analysis elaborates the data collection, where AIC evaluates the efficiency of individual models compared to other proposed models. Thus, the AIC considers a mean value for selecting a suitable model. The Bayesian information criterion (BIC) or Schwarz criterion (also SBC, SBIC) are well-known models in statistics for a finite set model selection. The most preferred model is that with the lower value of BIC that is dependent upon the likelihood function and is very close to the AIC.

The values of penalty functions like AIC and BIC totally depend upon the maximized value of likelihood function (L), which can be positive or negative. From the table above, we can see the lowest values were observed when the values of P, D and Q were one. We applied three models for load forecasting in the simulation, namely auto regression, artificial neural network, and ARIMA. In ARIMA, we have further applied the different models based on the P, D and Q values. The error rate from the neural network was 0.003, while from Auto Regression, it was 0.023. Based on these results, the best among them is ARIMA (1, 1, 1).

## 7. Conclusions and Future Work

This paper performed short-term load forecasting via three different prediction models and compared their accuracy. The results reveal that among the artificial neural network, auto regression, and ARIMA, ARIMA gave the best prediction with the highest accuracy. Therefore, from the prediction of different models of ARIMA, the model with (P, D, Q) value as (1, 1, 1) gave us the most accurate results. In the current study, we also visualized and analyzed the consumption patterns of ten different clusters. To identify the social, economic class of the customers and the various attributes that affect consumption patterns, such as holidays, weekends, temperature, family size, and work profiles, we evaluated the daily, weekly, monthly and seasonal power consumption. These assumptions and predictions can be helpful for utility companies in managing the demand response, asset management, investment in AMI, segmenting the customers, formulating the tariff plans. In the future, there will be an emphasis on using the ANN model or a combination of ANN with other models such as ARIMA and increasing the number of parameters for improvement of the model. It is obvious that one model is not efficient for all fields. Therefore, it is a constant effort to keep enhancing and choosing the model that fits our purpose. Therefore, there is a need to identify and define the capability of models for different predictions that give the fewest errors with very little variation for their estimations. Further, the analysis of the whole year and two or more seasons can be attained using parallel computing.

## Figures and Tables

**Table 1:** Results comparison.

Ref	Method	Results
[3]	Clustering	Entropy = 0.8056, Classification uncertainty = 10% for each customer
[7]	Kalman Filtering	Mean absolute percentage errors (MAPE) = 12.7058%
[9]	Regression, ANN	MAPE = 1.68%
[12]	ANN	Mean squared error (MSE) = 0.0012
[13]	ANN, ARIMA	ANN: MAPE = 22.25 ARIMA: MAPE = 696.86
[14]	ARIMA	Standard error = 1.82%

**Table 2:** Configuration to extract data for start and end time.

Value Column to Bin (1st Port)	Date_Time
Lower Bound Column (2nd Port)	b_time (#1)
Upper Bound Column (2nd Port)	e_time
Label Column (2nd Port)	in_range
If no rule matches	Insert missing
Search Pattern	Linear

**Table 3:** Electricity consumption by 10 clusters.

Day Segments	Hour
Early Morning	(7–9 h)
Morning	(9–13 h)
Early Evening	(13–17 h)
Late Evening	(17–21 h)
Night	(21–7 h)

**Table 4:** Datasets comparison.

Reference	Duration	Attributes	Pre-Processing
Proposed Study	3 years	10	K-means Clustering, Min–Max Normalization
[30]	1 year	5	K-means Clustering
[31]	1.5 years	81	Data Normalization
[32]	1 year	29	Feature Extraction
[33]	2 years	-	Data Filtering

**Table 5:** ARIMA results with different P, D and Q values.

ARIMA (P, D, Q)	011	021	022	100	110	111
AIC	AUD 932.289	AUD 903.531	AUD 887.843	AUD 1221.06	AUD 932.052	AUD 1208.46
SBC	AUD 920.9661	AUD 892.249	AUD 870.86	AUD 1209.72	AUD 920.724	AUD 1191.46

## Data Availability

Not applicable.
